# Linking observational data from general practice, hospital admissions and diabetes clinic databases: can it be used to predict hospital admission?

**DOI:** 10.1186/s12913-019-4337-1

**Published:** 2019-07-29

**Authors:** Sarah Dennis, Jane Taggart, Hairong Yu, Bin Jalaludin, Mark F. Harris, Siaw-Teng Liaw

**Affiliations:** 1 Faculty of Health Sciences, University of Sydney, 75 East Street, Lidcombe, NSW 2141 Australia; 2 Centre for Primary Health Care and Equity, University of New South Wales Australia, Sydney, NSW 2052 Australia; 3 Ingham Institute for Applied Medical Research, 1 Campbell Street, Liverpool, NSW 2170 Australia; 4 South Western Sydney Local Health District, Liverpool, Liverpool, NSW 2170 Australia; 5 School of Public Health and Community Medicine, University of New South Wales Australia, Sydney, NSW 2052 Australia

**Keywords:** Type-2 diabetes, Data linkage, Primary care, Hospital admission, Data quality

## Abstract

**Background:**

Linking process of care data from general practice (GP) and hospital data may provide more information about the risk of hospital admission and re-admission for people with type-2 diabetes mellitus (T2DM). This study aimed to extract and link data from a hospital, a diabetes clinic (DC). A second aim was to determine whether the data could be used to predict hospital admission for people with T2DM.

**Methods:**

Data were extracted using the GRHANITE™ extraction and linkage tool. The data from nine GPs and the DC included data from the two years prior to the hospital admission. The date of the first hospital admission for patients with one or more admissions was the index admission. For those patients without an admission, the census date 31/03/2014 was used in all outputs requiring results prior to an admission. Readmission was any admission following the index admission.

The data were summarised to provide a comparison between two groups of patients: 1) Patients with a diagnosis of T2DM who had been treated at a GP and had a hospital admission and 2) Patients with a diagnosis of T2DM who had been treated at a GP and did not have a hospital admission.

**Results:**

Data were extracted for 161,575 patients from the three data sources, 644 patients with T2DM had data linked between the GPs and the hospital. Of these, 170 also had data linked with the DC. Combining the data from the different data sources improved the overall data quality for some attributes particularly those attributes that were recorded consistently in the hospital admission data. The results from the modelling to predict hospital admission were plausible given the issues with data completeness.

**Conclusion:**

This project has established the methodology (tools and processes) to extract, link, aggregate and analyse data from general practices, hospital admission data and DC data. This study methodology involved the establishment of a comparator/control group from the same sites to compare and contrast the predictors of admission, addressing a limitation of most published risk stratification and admission prediction studies. Data completeness needs to be improved for this to be useful to predict hospital admissions.

## Background

In Australia, in 2014–15, there were approximately one million hospital admissions where diabetes was listed as a principal or additional diagnosis [1]. Admission for diabetes or diabetes related problems is a risk factor for further hospital admissions [2] and, in 2009–10, 24% of admissions for potentially preventable conditions were for diabetes [3].

A number of factors that increased the likelihood of admission for people with type-2 diabetes mellitus (T2DM) were identified using general practice (GP) quality of care data from the Quality and Outcomes Framework (QOF) and hospital admission data in the UK [4]. Good glycaemic control reduced the likelihood of admission but there were a number of potential confounders in that poorer glycaemic control was associated with disadvantage, which was also a risk factor for admission. Australian data from the CARDIAB diabetes registry found that records of care being provided, rather than targets such as glycated haemoglobin (HbA1c) achieved, were associated with a reduced risk of admission for people with diabetes [5]. However, aggressive management of HbA1c can increase the risk of admission with a study in the USA demonstrating a U-shaped relationship with HbA1c and hospital admission for cardiovascular events [6].

Linking process of care data from GP and hospital data may provide more information about the risk of hospital admission and re-admission for people with T2DM. Currently in Australia, organisations such as the New South Wales (NSW) Centre for Health Record Linkage (CHeReL) have routinely linked data from a wide range of sources but not often general practice data. Data from primary care disease registries has been linked to hospital data [5] and there are groups exploring the use of extraction tools such as GRHANITE™ to extract and link data [7] Linking data between general practice and hospital sources may result in a greater understanding of why some patients with T2DM are more likely to be admitted to hospital.

Australian health policy encourages general practices to use electronic health records to improve health outcomes for patients, especially for those with chronic conditions [8]. Increasingly, this data is being used for audit, continuous quality improvements and to evaluate the quality of care for people with chronic conditions with the assumption that the data is fit for purpose [8]. Health Information System (HIS) data in both hospitals [9] and GPs [10] are not adequate for clinical or health promotion purposes. Data quality (DQ) is “the totality of features and characteristics of a (data) entity that bears on its ability to satisfy stated and implied needs” (ISO 8402-1986, Quality Vocabulary). This “fitness for purpose” [11] definition is multidimensional and includes attributes such as “accuracy, perfection, freshness and uniformity” [12] and “completeness, unambiguity, meaningfulness and correctness” [13]. The Canadian Institute for Health Information promotes an information quality framework with six quality dimensions: accuracy, timeliness, comparability, usability, relevance and privacy & security [14]. DQ research has focused on generic core dimensions such as accuracy, currency and completeness [13], correctness, consistency and timeliness [15, 16]. More recently, a data quality framework has been proposed to standardise assessment and reporting of HIS data [17]. Linking data between GP and hospital sources may result in improved data quality in the combined dataset.

The University of New South Wales (UNSW) electronic Practice Based Research Network (ePBRN) was developed in the Fairfield local government area in South Western Sydney to conduct clinical and health services research, by extracting and linking routinely collected data from participating GPs and local health services including the hospital in the Fairfield Integrated Health Neighbourhood (FIHN) [10, 18]. The FIHN is a group of 10 GPs, Fairfield Hospital admissions, emergency department and diabetes clinic and the associated data repository which links and manages data extracted from the members. Development work during the establishment of the ePBRN indicated that improvements were required in the quality and accuracy of data entered in the HIS of both hospitals [9] and general practices [10].

The aim of this first proof of concept study was to extract and link data from a hospital, a diabetes clinic (DC) and GPs in the FIHN to determine if the linkage improves the quality of these data. A second aim was to determine whether the data could be used to predict hospital admission for people with T2DM.

## Methods

Ten GPs, Fairfield Hospital (FH) and the Diabetes Clinic (DC) at Fairfield Hospital were invited and consented to participate. All the GPs used Medical Director clinical software (https://www.medicaldirector.com). The DC is an outpatient clinic and takes referrals from both GPs and FH.

Data from each of the GPs, FH and DC were extracted separately using the GRHANITE™ extraction and linkage tool. This tool uses hash technology to mask a set of personal identifiers for use as a pseudonym so that patients cannot be identified in the extracted data [19]. This pseudonym is the basis for a unique patient identifier in each of the three datasets extracted from GPs, the DC and FH.

The date range of data extracted differed among the three sources (see Table 1), allowing us to capture information about a patient prior to a hospital admission. The data from GPs and the DC included data from the two years prior to the first hospital admission because two years would capture the annual cycle of care for diabetes patients [20]. The date of the first hospital admission for patients with one or more admissions was the index admission. Any subsequent admissions for the same patient for any reason were identified as readmissions. For those patients without an admission, the census date 31/03/2014 was used in all outputs requiring results prior to an admission or census date.

**Table 1:** Data source and summary of data extracted

Data Source and Date Range of Data	Data extracted
General Practice (GP) 01/01/07 to 31/03/14	Demographic characteristics (e.g. age, gender, ethnicity), risk factors (e.g. smoking, alcohol consumption, blood pressure), consultation information including Medicare Item Numbers, medical history (e.g. diagnoses), pathology results and prescriptions of all patients with a record at the practice regardless of a diagnosis of any type of diabetes.
Diabetes Clinic (DC) 01/01/07 to 31/03/14	Demographic characteristics, visit dates, who the patient saw (e.g. diabetes educator, endocrinologist), groups attended and pathology results for all patients with diabetes including T2DM, Type-1 Diabetes (T1DM) and Gestational Diabetes (GDM) who visited the Diabetes Clinic.
Fairfield Hospital (FH) 01/01/2009 to 31/03/14	Demographic characteristics, referral source, admission and discharge dates, readmissions, primary and up to 25 additional diagnoses, principal procedure, separations and transfers for all patients with any diabetes related diagnosis with an admission to Fairfield Hospital.

**Fig. 1:**
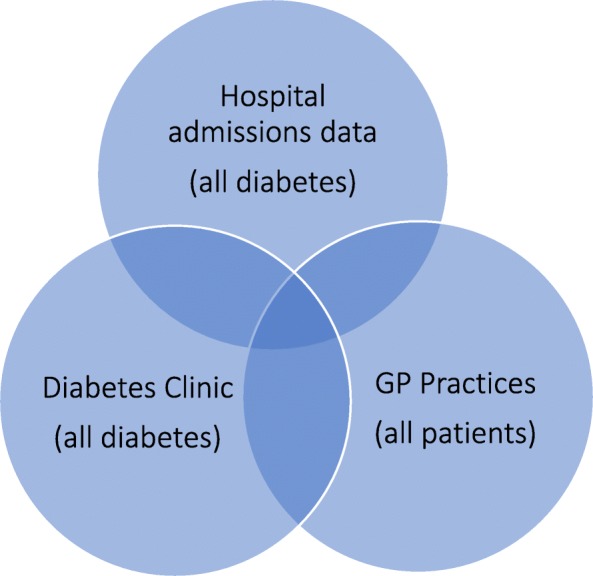
Illustration of the linkage of the data from the three sources

The extracted data were uploaded into the secure UNSW server and then moved into a study specific MS SQL™ database using the GRHANITE™ Databank Manager. The data from the three sources were linked as illustrated in Fig. 1.

The GRHANITE™ Linker Tool used five identifiers to link data from the three datasets:

- part of the Medicare number;
- date of birth;
- gender
- patient names; and
- postcode.

Linked records could be matched by up to four of the combinations of the above identifiers and more details can be found in the paper by Boyle et al. [21]. Identifying information was not extracted or provided to the researchers.

Once the data from the three sources had been linked by GRHANITE™, those with a diagnosis of T2DM were identified from the admissions data by the principal and any additional diagnoses recorded using the ICD-10 code for T2DM (E11) and from the general practice data using a validated algorithm that queried any T2DM diagnosis or reason for visit, a diabetes related prescription or an HbA1c result [18, 22]. Patients with T2DM in the diabetes clinic data could not be identified by diabetes type, as this information was not recorded electronically. The technical and qualitative assessment of conformance and plausibility of the data was extensive and conducted by two researchers (HY, JT) for missing, invalid or inconsistent gender, year of birth (YOB) and date of birth (DOB) to identify any linkages that may be false positive. Possible false positive links were excluded if they had been matched using only one combination of the identifiers in the linkage process and if there was no record of diabetes in the diagnosis, prescription or pathology record. Records linked with more than one combination of identifiers were included.

We extracted patient factors that have been shown to increase the risk of admission for T2DM, including patient demographics of age [4], [23, 24], gender [4, 24], ethnicity [4, 25]; risk factors such as obesity [26], smoking [24, 27], hypertension, hyperlipidaemia, glycaemia [4], [6, 26]; co-morbidities [23, 26, 28]; duration of diabetes [29] and process of care [5]. We identified patients with co-morbidities including cardiovascular disease (CVD), chronic kidney disease (CKD) and chronic obstructive pulmonary disease (COPD) from the GP data using diagnoses and prescriptions. CVD and CKD were included to capture patients with complications from T2DM.

HbA1c results were categorised as controlled (≤7%; ≤53.0 mmol/mol), uncontrolled (> 7% and < =8%; > 53.0 and < =63.94 mmol/mol) and very uncontrolled (> 8%; > = 63.94 mmol/mol). We also collected information on prescriptions for diabetes, CVD, lipid lowering medications, antihypertensives, low dose aspirin and anticoagulants, antiarrhythmics, beta blockers, anti-anginal medications, COPD and CKD. The list of medications was identified from the literature and best practice guidelines [20] for the management of T2DM patients.

The data were summarised to provide a comparison between two groups of patients:

1. Patients with a diagnosis of T2DM who had been treated by GPs in the eBPRN and had a Fairfield Hospital admission.
2. Patients with a diagnosis of T2DM who had been treated by GPs in the eBPRN and did not have a Fairfield Hospital admission.

Following the extensive qualitative examination to verify the conformance and plausibility of the data. Descriptive statistics were used to summarise and determine the completeness of the linked data. To explore whether the data could be used to identify risk factors that predict hospital admission, we used multiple logistic regression analysis to identify if there was an association between selected variables and risk of hospital admission. Chi-squared was used to assess the association between the individual attributes and the admission status in the univariate analysis. All attributes where the *p*-value was < 0.20 in the univariate model were included in the multivariate regression model. The backward selection method was used to derive the final predictive model. For medication and GP/DC visits, the results are presented for each unit increase from baseline and for medication for each additional medication prescribed. The completeness of the data determines if the data are sufficient for modelling. The outcome of the modelling is compared to the published literature to determine the plausibility.

Ethics approval was obtained from South Western Sydney Local Health District Human Research Ethics Committee (HREC/10/LPOOL/29) and ratified by the ethics committee of the University of New South Wales.

## Results

**Fig. 2:**
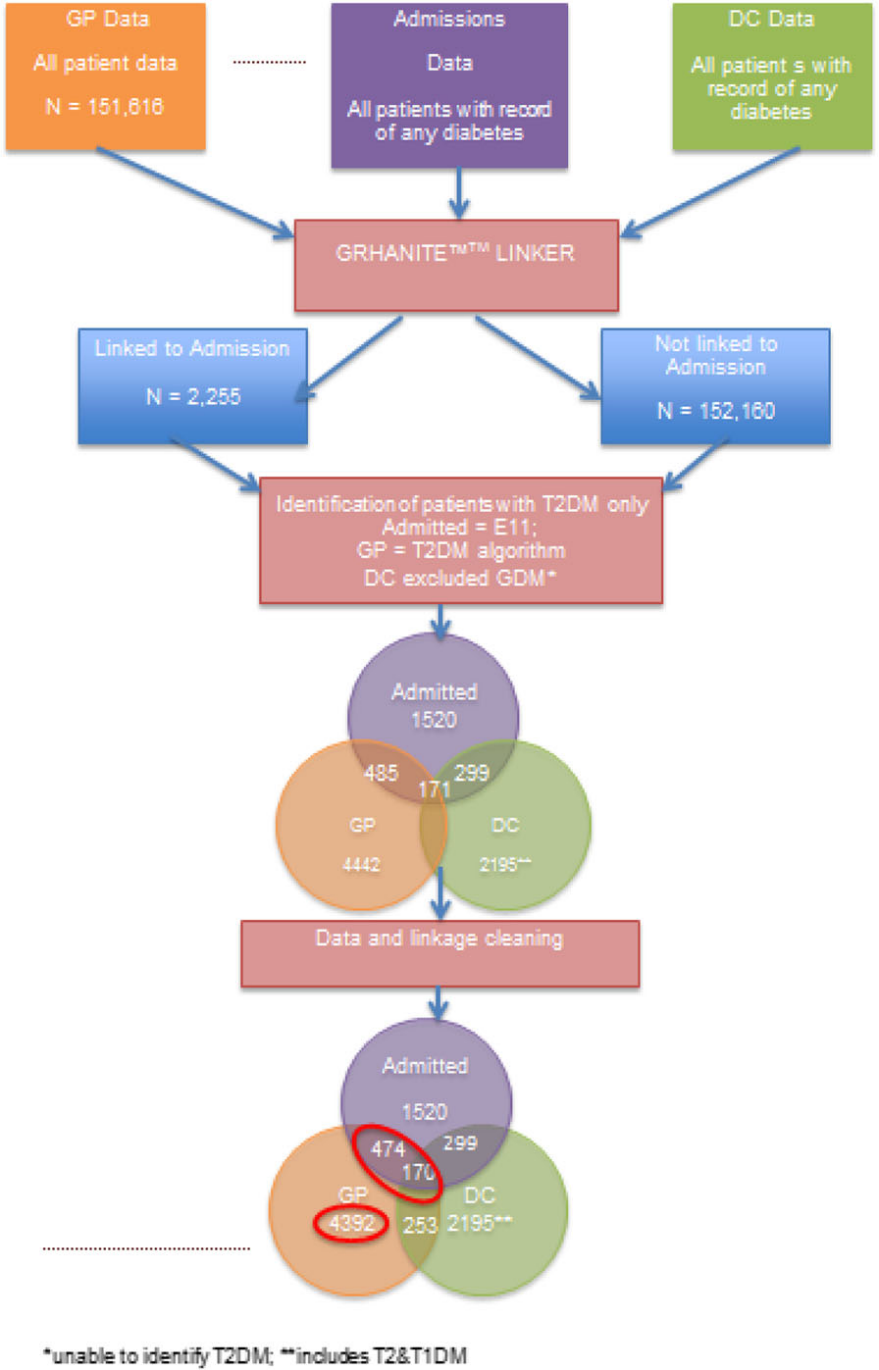
Flow chart to illustrate the process of data extraction and data cleaning

All ten general practices in the ePBRN, Fairfield Hospital and the Diabetes Clinic agreed to take part in the study. However, due to a technical issue, one of the general practices could not be included, so data were only extracted from the remaining nine practices. Data were extracted for a total of 161,575 patients from the three data sources. There were 644 patients with T2DM whose hospital records were able to be linked to the GP dataset and, of these, 170 patients also had records that could be linked to the DC dataset (Fig. 2).

There was variation in data quality between attributes and between data sources. Combining the data from the different data sources improved the overall quality for some attributes (Table 2), generally those attributes that were recorded consistently in the hospital admission data. For example, demographic data were 100% complete in the admissions and Diabetes Clinic data while in the GP data marital status (45.5%), Aboriginal and Torres Strait Islander status (50.5%) and country of birth (6.9%) were often not recorded. This meant there were lower rates of completeness of demographic information for patients without a hospital admission or Diabetes Clinic visit, except for age and gender, which were recorded in 100% of the GP records. Smoking, alcohol consumption, and body mass index (BMI) were sourced from the GP data only and they ranged from 2.9% complete for alcohol consumption to 64.5% for smoking status (Table 2).

**Table 2:** Completeness of combined data from general practices, Fairfield Hospital and Diabetes Clinic for admitted and not admitted patients

Attributes	With admission (*n* = 644) *N*(%)	Without admission (*n* = 4,392) *N* (%)	All patients (*n* = 5,036) *N* (%)
Year of birth (YOB)	644 (100)	4,392 (100)	5,036 (100)
Gender	644 (100)	4,392 (100)	5,036 (100)
Marital status	644 (100)	4,071 (92.7)^a^ 1,998 (45.5) ^b^	4,715 (93.6)^a^ 2642 (52.5) ^b^
Aboriginal & Torres Strait Islander	644 (100)	2,218 (50.5)	2,862 (56.8)
Country of Birth (COB)	644 (100)	303 (6.9)	947 (18.8)
Body Mass Index (BMI)	177 (27.5)	1,495 (34)	1,672 (33.2)
Smoking status	342 (53.1)	2909 (60.2)	3,251 (64.5)
Alcohol consumption	27 (4.2)	121 (2.8)	148 (2.9)
Any pathology results	373 (57.9)	2223 (50.6)	2596 (51.5)
HbA1c	290 (45)	2,034 (46.3)	2,324 (46.1)
Total Cholesterol	302 (46.9)	2,052 (46.7)	2,354 (46.7)
HDL-Cholesterol	248 (38.5)	1,871 (42.6)	2119 (42.1)
LDL-Cholesterol	252 (39.1)	1,843 (42)	2,095 (41.6)
Triglycerides	301 (46.7)	2,052 (46.7)	2,353 (46.7)
Urinary albumin	81 (12.6)	345 (7.9)	426 (8.5)
Systolic Blood Pressure	413 (64)	3,342 (76.1)	3,755 (74.6)
All prescriptions	644 (100)	4,344 (98.9)	4,988 (99.0)
Prescriptions -T2DM related	344 (53.4)	3,588 (81.7)	3,932 (78.1)
Prescriptions – Not T2DM related	555 (86.2)	4,344 (98.9)	4,899 (97.3)
GP Visits	644 (100)	4,392 (100)	5,036 (100)
Diabetes Clinic visits	170 (26.4)	334 (7.6)	504 (10.0)
Co-morbidities total#	393 (61)	245 (5.6)	638 (12.7)
Cancer	48 (7.5)	262 (6)	310 (6.2)
Psychological	146 (22.7)	72 (1.6)	218 (4.3)
Macrovascular	330 (51.2)	200 (4.6)	530 (10.5)
Microvascular	81 (12.6)	38 (0.9)	119 (2.4)
Metabolic disorders	15 (2.3)	15 (0.3)	30 (0.6)
Overweight/obesity related	127 (19.7)	84 (1.9)	211 (4.2)
Other	31 (4.8)	38 (0.9)	69 (1.4)
CVD	400 (62.1)	2,691 (61.27)	3,091 (61.4)
CKD	125 (19.4)	263 (5.9)	388 (7.7)

“With admission” includes GP, DC and hospital data, “without admission” includes GP and DC data only

^a^2073 coded as “Unknown”, ^b^ with the 2073 “Unkown” removed

**Fig. 3:**
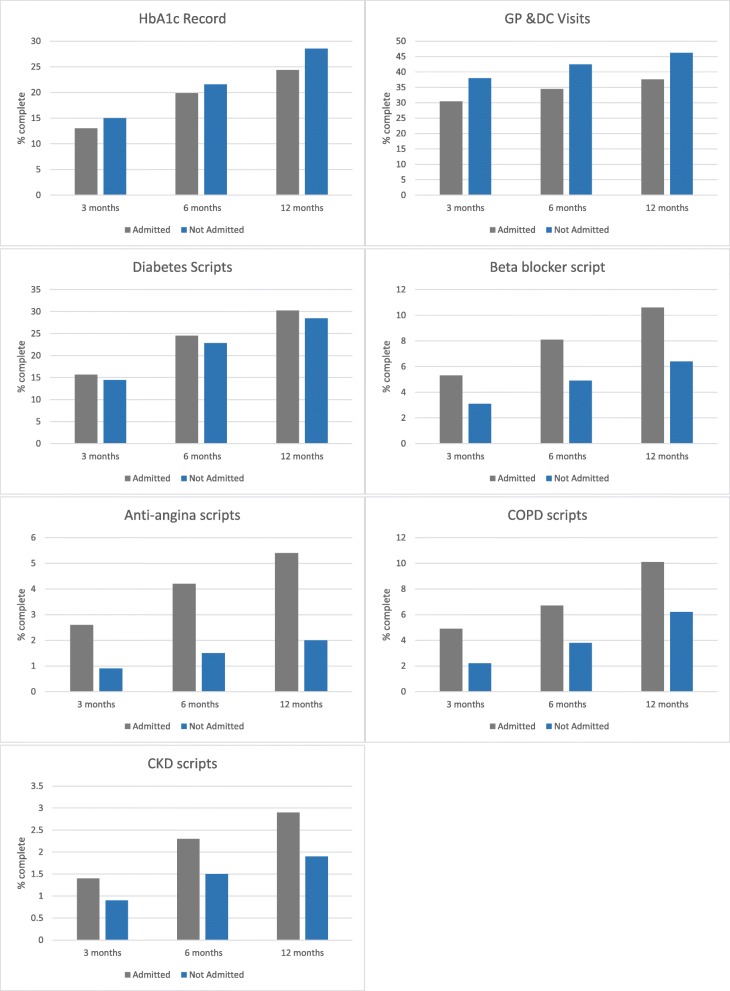
Completeness of a selection of attributes by time prior to admission or census date

Pathology results were available from the GP data and Diabetes Clinic data for 57.9% of patients with an admission and 50.6% for those without an admission and there was variation in the proportion of patients with specific results. HbA1c results were available for 45% of patients with an admission and 46.3% of patients without an admission while urinary albumin excretion results had the lowest completeness rate of 12.6 and 7.9% respectively. All patients, whether they had an admission or not, had records for diagnoses and almost all patients had prescription records in the GP data (100% for admitted and 98.9% for not admitted). When temporal dimensions were added to the analysis to explore whether there were trends over time, data completeness was reduced further (Fig. 3). This reduction in data completeness limited the potential to analyse trends in pathology results over time prior to an admission that might be a useful predictor of admission.

The proportion of GP visit records that included the visit date were higher for admitted patients than non-admitted patients (98.4 and 82.7% respectively). The records without a visit date all came from the same practice, which may indicate a problem within their clinical record system, in the extraction of the data or in data management in the data repository. Visit dates were available for all Diabetes Clinic visits.

The demographic and clinical characteristics of the patients with T2DM with a hospital admission and no admission are listed in Table 3. As expected patients with a hospital admission and linked GP data tended to be older (*p* < 0.0001) and they were in poorer health with a higher prevalence of CKD (p < 0.0001) and CVD (*p* = 0.009) compared to those GP patients with T2DM without an admission to hospital.

**Table 3:** Demographic and clinical characteristics of the patients with T2DM

	Linked Admitted Patients with T2DM (N = 644)		General Practice Patients with T2DM & No Admission (*N* = 4392)	
	Number	%	Number	%
Females	342	53.1	2,283	51.9
Age group (years)				
< 20	0	0	25	0.6
20–44	32	4.9	492	11.2
45–64	177	27.5	1,720	39.2
65–74	162	25.2	1,030	23.5
≥ 75	273	42.4	1,125	25.6
Mean (SD) age in years#	69.4 (12.9)		63.4 (15.9)	
Smoker	49	7.6	438	10.0
GP Diagnosis of Cardiovascular disease (CVD)*	322	50	2,438	55.5
GP Diagnosis of Kidney disease (CKD)#	53	8.2	158	3.6
GP Diagnosis of Chronic obstructive pulmonary disease (COPD)	95	14.8	555	12.6
Mean (SD) number of GP & DC visits^	6 (13.1)		6.3 (10.9)	
Median (range) number of GP & DC visits^	0 (0, 90)		0 (0, 130)	
Mean (SD) HbA1c^	7.25 (1.5)		7.35 (1.6)	
Median (range) number of HbA1c recorded	4 (0, 28)		3 (0, 25)	
Number with readmission at Fairfield Hospital	252	39	N/A	N/A

#p < 0.0001, *p = 0.009, ^within 12 months prior to admission or census date

There were 170 (26.4%) patients with a hospital admission and a record of attendance at the Diabetes Clinic compared to only 7.6% for those patients without a hospital admission. Whilst a greater proportion of the patients admitted to hospital had also attended the Diabetes Clinic for management of their T2DM, the mean number of visits was similar between the two groups (see Table 3).

The results of the logistic regression analysis are presented in Table 4. Those patients who were older were more likely to be admitted to hospital, as were those who were prescribed three or more medications to manage their hypertension or one or more medications to manage COPD and those with a GP recorded diagnosis of chronic kidney disease. A moderate number of visits to the GP or DC seemed to reduce the risk of admission but patients were more likely to be admitted as the number of visits became very high, which suggests the patient might be sicker. There were mixed results for HbA1c to predict hospital admission and CVD was not identified as a risk factor. This is not unexpected given the problems with the completeness of the data for HbA1c and CVD related drugs illustrated in Table 2 and Fig. 3.

**Table 4:** Associations between socio-demographic and clinical characteristics and hospitalisation for type-2 diabetes from a multivariable regression model

	Odds Ratio	95% CI
Age group (years)		
• 0–44 (reference)	1.0	
• 45–64	1.94	1.30–2.88
• 65–74	2.99	1.99–4.52
• 75+	4.26	2.85–6.38
Smoking status		
• Non-smoker (reference)	1.0	
• Ex-smoker	1.49	1.15–1.93
• Smoker	1.37	0.96–1.94
• Missing value	1.82	1.47–2.27
Number of visits to general practice/Diabetes Clinic		
• 0 (reference)	1.0	
• 1–5	1.14	0.89–1.47
• 6–20	0.44	0.33–0.59
• 21–30	0.66	0.44–0.99
• 31+	0.70	0.45–1.09
Number of antihypertensive medications		
• 0 (reference)	1.0	
• 1	1.6	0.75–1.48
• 2	1.26	0.86–1.85
• 3+	3.12	2.15–4.52
Low dose aspirin		
• 0 (reference)	1.0	
• 1+	1.59	1.12–2.27
Number of COPD medications		
• 0 (reference)	1.0	
• 1+	1.50	1.08–2.09
HbA1c category		
• ≤7% (Controlled diabetes) (reference)	1.0	
• > 7% and ≤ 8% (Uncontrolled diabetes)	2.09	1.38–3.15
• > 8% (Very uncontrolled diabetes)	1.48	0.93–2.35
• Missing value	1.44	1.08–1.93
GP diagnosis of cardiovascular disease (CVD)	0.54	0.44–0.66
GP diagnosis of chronic kidney disease (CKD)	1.98	1.38–2.84

## Discussion

This study has demonstrated that linking observational data across the nine general practices, Fairfield Hospital and the Diabetes Clinic that make up the *Fairfield Integrated Health Neighbourhood* (FIHN) is feasible. However, there are issues with data quality for some variables. The strength of this methodology was the ability to easily identify a comparator/control group, in this case admitted versus non-admitted patients in the GP population. It strengthened the predictive model in so far as we were able to demonstrate that those patients with a hospital admission were older, in poorer health and with comorbidities identifying potential predictors for admission from the data and verified the data. It was both a strength and a weakness that the practices all used the same practice software. At this proof of concept stage it reduced any differences that might have occurred if data were recorded or extracted inconsistently from other practice software. However, if extracting and linking data in this way are to be useful then the approach must be demonstrated to work for other general practice software. There are limitations particularly with respect to the quality of the observational data for modelling and research and whilst the results of the predictive model made clinical sense they should be treated with caution.

There were expected problems with data quality from the three data sources. Nevertheless, linking the data sources did improve data quality by increasing the completeness of the data. It has the potential to be useful to identify the factors that are associated with hospital admission for patients with T2DM and to identify general practice patients at risk of admission to hospital.

To be able to identify patients at risk and share clinical information with providers to improve patient outcomes and to be useful in clinical practice, there is an urgent need to improve the data quality, especially the completeness. We examined the data completeness issues at the general practice level. Two of the practices in particular had low completeness across most of the data attributes compared with the other practices. One of these practices was relatively new and one of the doctors at the practice preferred to write in the clinical notes instead of entering clinical data into the defined fields of the database. The GPs at the other practice were less engaged with the ePBRN intervention although they had made some improvements in data completeness [30]. All practices and the diabetes clinic were provided with the results of the study and regular structured data quality reports every six months during this study [30]. They were encouraged to use these reports to reflect on their data quality and identify opportunities for improvement. In addition to this the quality improvement activities are ongoing through the Australian Primary Care Collaboratives Program, the Royal Australian College of General Practitioners (RACGP) and the primary care organisations make use of the PENCat audit tool to assist with improving data quality and to encourage clinicians to enter data in structured fields [31].

We have previously found the ePBRN general practice data quality was consistent with other studies [10]. Improving data quality requires a comprehensive sociotechnical approach. The technical dimensions need to address the user interface to support data collection during the work flow that include drop-down menus and decision support tools to promote structured data entry using standard terminology and coding. Differences in practice software have been shown to impact on the quality of data [32]. The social aspects need to address the issues at the individual, organisation and health system levels. A recent paper identified steps involved in recording data in the practice software which included: 1) was the test performed? 2) if performed was it recorded? 2) where was it recorded? and was it extracted? [33]. In this study the data needed to be entered into the database fields and not as free text in the notes section to be extracted. Studies have demonstrated that providing practices with feedback on their data quality and discussions around potential solutions to improve local data quality are effective [30, 32]. This highlights the need for continuing education, support and feedback to GPs and practice staff to record patient information accurately, completely and in the appropriate place and to make best use of the HIS in supporting safe and effective clinical care. The utilisation of computerised medical records in general practice has been slower in Australia and 2006 data indicated that only a third of GPs used a computer for all their patient information [34]. A more recent study in Australian integrated primary health care centres [35] found that HIS data extracted did not meet the RACGP guidelines standards for general practice [31]. The hospital and diabetes clinic data had high rates of completeness for the administrative data. Hospitals have key staff and processes in place for entering hospital admission and administrative information which may have contributed to the higher data completeness. The implementation of electronic clinical records at the DC could also improve the quality of the linked data, particularly in relation to diabetes type and risk factors.

Evidence from the introduction of the Quality and Outcomes Framework in the UK has shown that data detailing general practice process of care measures for the management of diabetes has increased with financial incentives [36]. Similar improvements in the recording of diagnosis codes has been seen following payments to GPs to address data quality in the Netherlands [37]. There have not been similar incentives to improve data quality in Australian general practices although there have been some improvements in data quality with the Australian Primary Care Collaboratives Program [38]. Including patients as part of the strategy to improve data quality may also be worth exploring. The Collaboratives Program found patients were ‘effective allies’ when involved in checking the accuracy of their clinical records to assist practices to improve data quality. [39]. A recently completed study that explored eHealth initiatives and health care integration in integrated primary health care centres recommended the development of internal structures and protocols to promote improvements in data quality and a national approach to audit, feedback, continuous quality improvement, research and outcomes monitoring to support and promote a culture that values good data and documentation [40]. We are also exploring the use of ontological methods which are automated approaches to address and improve data quality at the repository end. Research in the use of an ontological approach is growing [41–46]. We have successfully used this approach in developing the algorithm to identify the T2DM patients in this study.

The regression analysis confirmed that the data completeness for key clinical and demographic characteristics impacted on the ability to be useful to predict hospital admission. Substantial work needs to be undertaken to increase the data quality for the characteristics identified in the literature as increasing the likelihood of hospital admission. However, there were some findings that are consistent with other studies. The number of people who were identified as Aboriginal or Torres Strait Islander in this study was too small and was not included in the model but has been found to be a risk factor in other studies [3]. Our population of people with T2DM was drawn from an area of very high ethnic diversity, with 68.4% of the population born overseas [47]. The area is classified as low socioeconomic status [47] and the data extracted to identify this, such as postcode, was not included in the model. The lack of a clear association with very poorly controlled HbA1c and the protective effect of GP visits was consistent with the CARDIAB results where the process of care might be more important than the actual value for HbA1c [5]. The data missingness for the HbA1c data might explain why we did not demonstrate a U-shaped relationship between HbA1c and hospital admission found by other groups [6].

The main limitations of this study were the small number of general practices used and the leakage from the FIHN. The general practices were located in close proximity to Fairfield Hospital but there are two other large teaching hospitals within 15 and 30 km from Fairfield Hospital where patients might have been admitted and data from these hospitals was not extracted as part of this study. In Australia, GPs operate in a fee for service system and patients are not registered with a particular GP. This means that patients can visit multiple GPs for their care. Patients may also have visited other general practices not included in this study. However, this study has demonstrated that the linkage and cleaning processes that have been established make it feasible to extend the data extraction and linkage to other hospitals and general practices. This would increase the numbers of patients and reduce the leakage of patients to other hospitals thereby improving the robustness of the model. A study is now under way in the area that includes six hospitals, one of which is a major teaching hospital, and 15 general practices. A further limitation is a focus on completeness as a component of data quality. This is an important component of data quality, as is verification, validation and the temporal dimension which were also considered in this study [17].

## Conclusion

The Fairfield Integrated Health Neighbourhood (FIHN) project has established the tools and processes to extract, link, aggregate and analyse observational data from general practices, hospital admission data and Diabetes Clinic data. This study methodology involved the establishment of a comparator/control group from the same IHN to compare and contrast the predictors of admission, addressing a limitation of most published risk stratification and admission prediction studies.

In addition to confirming the known clinical predictors, the regression analysis also suggests that continuity of care with a provider might protect against admission. The data “missingness” did not permit an exploration of predictors of re-admission. The data quality, data completeness in particular, must be improved for predictive modelling to be useful.

The recent policy announcement relating to the “Health Care Home” [48] and some form of patient enrolment will address some of the leakage from the Fairfield Health Neighbourhood (FIHN), and enable the evaluation of this policy direction. In addition, this work positions the FIHN to support ongoing longitudinal cohort studies, using observational data, in a range of domains to answer a range of health services and population health questions.

## Data Availability

The datasets generated and/or analysed during the current study are not publicly available due to the limits of the ethics approval but are available from the corresponding author on reasonable request.

## Abbreviations

BMI: Body mass index
CHeReL: Centre for Health Record Linkage
CKD: Chronic kidney disease
COPD: Chronic obstructive pulmonary disease
CVD: Cardiovascular disease
DC: Diabetes Clinic
DOB: Date of birth
DQ: Data quality
ePBRN: Electronic Practice Based Research Network
FH: Fairfield Hospital
FIHN: Fairfield Integrated Health Neighbourhood
GP: General practice
HbA1c: glycated haemoglobin
HIS: Health Information System
NSW: New South Wales
QOF: Quality and Outcomes Framework
T2DM: Type-2 diabetes mellitus
UNSW: University of New South Wales
YOB: Year of birth
